# Quantitative characterization of agglomerates and aggregates of pyrogenic and precipitated amorphous silica nanomaterials by transmission electron microscopy

**DOI:** 10.1186/1477-3155-10-24

**Published:** 2012-06-18

**Authors:** Pieter-Jan De Temmerman, Elke Van Doren, Eveline Verleysen, Yves Van der Stede, Michel Abi Daoud Francisco, Jan Mast

**Affiliations:** 1 Electron Microscopy-unit, Veterinary and Agrochemical Research Centre (CODA-CERVA), Groeselenbergstraat 99, Brussels, 1180, Belgium; 2 Unit for Coordination of Veterinary Diagnostics, Epidemiology and Risk Analysis (CVD-ERA), Veterinary and Agrochemical Research Centre (CODA-CERVA), Groeselenbergstraat 99, Brussels, 1180, Belgium

## Abstract

**Background:**

The interaction of a nanomaterial (NM) with a biological system depends not only on the size of its primary particles but also on the size, shape and surface topology of its aggregates and agglomerates. A method based on transmission electron microscopy (TEM), to visualize the NM and on image analysis, to measure detected features quantitatively, was assessed for its capacity to characterize the aggregates and agglomerates of precipitated and pyrogenic synthetic amorphous silicon dioxide (SAS), or silica, NM.

**Results:**

Bright field (BF) TEM combined with systematic random imaging and semi-automatic image analysis allows measuring the properties of SAS NM quantitatively. Automation allows measuring multiple and arithmetically complex parameters simultaneously on high numbers of detected particles. This reduces operator-induced bias and assures a statistically relevant number of measurements, avoiding the tedious repetitive task of manual measurements. Access to multiple parameters further allows selecting the optimal parameter in function of a specific purpose.

Using principle component analysis (PCA), twenty-three measured parameters were classified into three classes containing measures for size, shape and surface topology of the NM.

**Conclusion:**

The presented method allows a detailed quantitative characterization of NM, like dispersions of precipitated and pyrogenic SAS based on the number-based distributions of their mean diameter, sphericity and shape factor.

## Background

The regulatory definition of a NM was and is an issue of debate [1-4], but it is agreed that a NM contains a relevant fraction of unbound, aggregated or agglomerated particles with one or more external dimensions in the size range of one to 100 nm. These particles are minute pieces of matter with defined physical boundaries [2,5]. For aggregates and agglomerates, these particles are referred to as primary particles [6]. The physical and chemical properties of a NM may be different from the properties of the corresponding bulk material because of quantum and surface effects which are size dependent [7]. The effects of a NM on an organism or cell depend on the characteristics of its aggregates and agglomerates, as well as on the size of its primary particles [8,9]. The size of aggregates and agglomerates of NM but also their morphology and their charge, coating and reactivity of their surface were shown to influence their interactions with biological systems [3,4,10-16].

The primary particles of pyrogenic and precipitated amorphous silica tend to aggregate and agglomerate during the production processes [17,18]. Pyrogenic or fumed silica is formed by reaction of water vapor produced by a hydrogen-oxygen flame with silicon tetrachloride to produce small, essentially spherical primary particles which subsequently collide to form rigid, covalently bound aggregates [19]. Precipitated silica is formed by destabilization and precipitation of an alkaline silicate solution [20]. Such SAS aggregates and agglomerates have fractal-like characteristics. The fractal dimensions of these complex three-dimensional nano-objects can be computed from two-dimensional (2D) TEM micrographs [21] or from testing the small-angle X-ray scattering (SAXS) data using a fractal geometry concept [22,23].

The yearly European production of pyrogenic and precipitated silica in year 2000 was 73,900 and 337,100 tons respectively, while the European consumption of these SAS was 368,000 metric tons [24]. SAS have a widespread use, giving raise to general human (and environmental) exposure, and are applied as additives to cosmetics, drugs, printer toners, paints and varnishes, car tires and food [25,26]. Many aspects related to the size of SAS have raised concerns about safety [27]. The unique physicochemical properties of nano-sized silica that make it attractive for industry may present potential hazards to human health, including an enhanced ability to penetrate intracellular targets in the lung and systemic circulation [20].

The size, physical form and morphology of NM can be investigated by electron microscopy methods. Image analysis techniques allow on one hand the direct visualization of NM and on the other hand, the analysis of the size, elongation, curvature of the particle corners and smoothness of the particle surface [28-31]. This paper presents a quantitative method to assess the characteristics of agglomerated and aggregated NM, exemplified by SAS. BF-TEM is combined with systematic random imaging and semi-automatic image analysis to obtain an accurate and representative quantification. In addition to the size of nano-structured agglomerates and aggregates, their morphology and surface structure are analyzed. To explore the possibilities of this methodology, examples of precipitated and pyrogenic silica NM in their most dispersed form are analyzed and compared as model systems.

## Methods

SAS NM-200, NM-201, NM-202 and NM-203 were obtained from the NM repository of the European Commission Joint Research Centre, Institute for Health and Consumer Protection, (JRC-IHCP, Ispra, Italy). Their respective BET values are 230, 160, 200 and 226 m²/g [32]. NM-200 and NM-201 are produced by precipitation and NM-202 and NM-203 are pyrogenic, and all are available as dry powders. These powders were suspended in double distilled water at a concentration of 2.56 mg/ml and sonicated for 16 minutes using a Vibracell™ 75041 ultrasonifier (750 W, 20 kHz, Fisher Bioblock Scientific, Aalst, Belgium) equipped with a 13 mm horn (CV33) at 40% amplitude. This setup resulted in an average horn power of 26 W and a sample specific energy of 2530 ± 20 MJ/m³. During sonication the samples were cooled in water with ice to prevent excessive heating. After sonication, the samples were diluted to a concentration of 0.512 mg/ml. The obtained dispersions were stable for at least two hours: no visible precipitates were observed.

By the grid on drop method, the suspended NM were brought on pioloform- and carbon-coated, 400 mesh copper grids (Agar Scientific, Essex, England) that were pretreated with 1% Alcian blue (Fluka, Buchs, Switzerland) to increase hydrophilicity as described in [33].

The samples were imaged in BF mode using a Tecnai G^2^ Spirit TEM (FEI, Eindhoven, The Netherlands) with Biotwin lens configuration operating at 120 kV mounted with a condenser aperture of 100 μm and an objective aperture of 150 μm. The condenser lens current was chosen such that the beam was parallel and images were taken approximately 500 nm below minimal contrast conditions, where Fresnel fringes were minimal and contrast was judged to be optimal.

To avoid subjectivity in the selection of particles by the microscopist, micrographs were taken randomly and systematically, at positions pre-defined by the microscope stage and evenly distributed over the entire grid area. When the field of view was obscured, *e.g.* by a grid bar or an artifact, the stage was moved sideways to the nearest suitable field of view. For each NM three independent samples were analyzed. Per sample, five micrographs were recorded with a 4*4 k Eagle CCD camera (FEI, Eindhoven, the Netherlands) at a magnification of 18,500 times using the TEM imaging *&* analysis (TIA) software (FEI, Eindhoven, The Netherlands). These SER- and EMI- formatted micrographs were converted to TIFF format using TIA. For the given microscope and camera configuration, this magnification results in micrographs with a pixel size of 0.60 nm and a field of view of 2.45 μm by 2.45 μm. This implies a lower particle size detection limit of approximately 6 nm, supporting on the criterion of Merkus [34] that large systematic size deviations can be avoided if the particle area is at least hundred pixels. The field of view restricts the upper size detection limit to 245 nm, one tenth of the image size as recommended [35]. The useful range is defined by the lower and upper size of the detection limit. To estimate the number of particles required for the estimation of the mean particle diameter with a confidence level, it is assumed that the size distribution can be approximated by a log-normal distribution. The minimal number of particles can then be calculated according Matsuda and Gotoh [35,36].

To achieve maximum traceability of information, each micrograph was stored with its administrative and sample preparation information as well as the information related to its imaging conditions in a dedicated database integrated in the iTEM software (Olympus, Münster, Germany). At several levels, modifications of the TIA software and of the iTEM software were made to transfer the micrographs and their associated microscope data efficiently into the iTEM database. (i) The TIA protocol for batch conversion of the software-specific SER- and EMI-formats was adjusted to avoid too long file names. (ii) An imaging C- and libtiff library-based module, referred to as the Tia-Tag module, was developed in iTEM. This module reads the information relevant for image analysis and quality control in the private tags of the TIF image files and renders it accessible in a new information tab of the iTEM software. In addition, the Tia-Tag module facilitates calibration of images by automatically converting the pixel size from mm scale to nm scale. (iii) New fields were defined in the iTEM database specifying the sample and sample preparation characteristics. Where applicable, drop lists were developed to avoid typographical errors.

In addition to the micrograph related information, the annotated images obtained during image analysis and the results and reports of these analyses were stored in the database, linked to the original micrograph.

The ‘iTEM solution detection’ was used for threshold-based detection of the NM. Briefly, the contrast and brightness of the micrographs were optimized, a 10 x 10 smoothing filter was applied, the involved particles were enclosed in a pre-defined frame or region of interest and thresholds were set to binarize the image and to separate particles from the background based on their electron density and size. Particles with an area of less than fifty pixels and particles on the border of the frame were omitted from analysis.

For each particle, twenty-three quantitative parameters, selected in the ‘Define measurements dialog box’ of the ‘iTEM solution detection’ and described in Additional file 1, were measured and considered relevant for its characterization.

Each particle detected in a micrograph was identified by a unique number, written in the overlay of the image. This allowed the selection of data of individual particles and the post-analysis deletion of erroneously detected particles. In general, artifacts were characterized by their morphology and a grey value lower than the mean grey value of the background plus three times its standard deviation. Particles fulfilling this criterion were identified and deleted automatically. Particles with an unusual morphology, judged to be artifacts based on visual inspection of the micrographs, were omitted manually from analysis.

The results obtained for each micrograph were combined in a data sheet in XLS-format format (Excel, Microsoft, Redmond, Washington, USA). This XLS-file was introduced in Sigmaplot (Systat, Cosinus Computing, Drunen, the Netherlands) and in the SAS statistical software (SAS Institute Inc., Cary, NC, USA). Descriptive statistics and histograms were calculated in Sigmaplot. The normality of the distributions of the measured parameters was tested with the Shapiro-Wilk and the Kolmogorov-Smirnov tests, while the homogeneity of variances was tested with the Spearman rank correlation test. Since these assumptions were not met, the non-parametric Kruskal-Wallis one-way ANOVA was performed and data were compared pairwise with the Dunn’s Method to determine the micrograph and sample effects, and to determine the effect of sonication on the number of particles per grid area. The normality of the distributions and the homogeneity of variances were met for the mean values of the median of the mean diameter, the median sphericity and the median shape factor of the different silica NM that were obtained in independent analyses. Hence, a one-way analysis of variance (ANOVA) was performed and data were compared pairwise with the Tukey test. The measured parameters were classified by PCA using the SAS statistical software.

## Results

### Sample preparation

**Figure 1:**
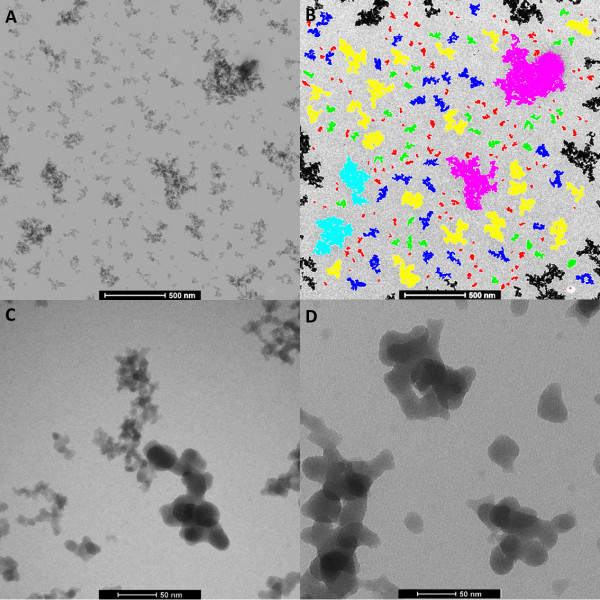
**Illustrations of the detection of silica NM-201 based on electron density and of the primary particles of NM-201 and NM-203.** The NM in the representative electron micrograph ( **A**) are detected, classified by mean diameter and false colour-coded in the corresponding annotated image ( **B**). Red: < 50 nm, green: 50–70 nm, blue: 70–100 nm, yellow: 100–200 nm, cyan: 200–300, pink: 300–500 nm and brown: > 500 nm. Particles at the borders of detection region are black and are omitted from analysis. Bar 500 nm. The selected electron micrographs illustrate the differences in primary particle size between NM-203 ( **C**) and NM-201 ( **D**). Bar 50 nm.

By adjusting the charge of the grid, the attachment of the negatively charged silica NM to the EM grid could be assured (Figure 1). Alcian blue pretreatment introduced positive charges on the surface of pioloform- and carbon-coated grids that tend to have a negative or neutral charge. In our experience, this approach is easier than the alternative based on glow discharging EM-grids with air [37] to introduce negative charges and subsequent Mg^2+^ treatment, introducing positive charges.

**Figure 2:**
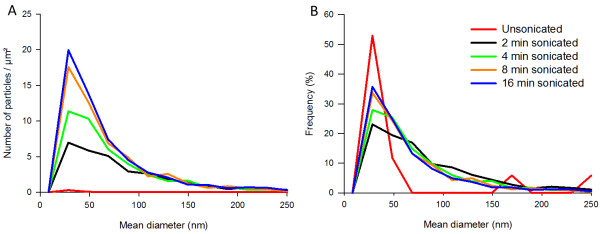
**Number-based size distributions visualising the effect of sonication on the size distribution of the precipitated silica NM-200.** The number of particles per μm² of grid area for a concentration of 0.512 mg/ml ( **A**) and the corresponding frequencies ( **B**) for unsonicated (Red), 2 min (Black), 4 min (Green), 8 min (Orange) and 16 min (Blue) of sonication, are represented as a function of their mean diameter.

To obtain homogenous and stable suspensions and a sufficient number of particles per grid surface, the examined silica NM required sonication and dilution. The number of NM-201 particles per grid area increased with sonication time (Figure 2). For eight and 16 minutes of sonication, the total number of detected aggregates was 1564 and 1674, respectively. This was higher than 1366, the number of particles allowing an estimation of the geometric mean particle size with an error of maximum five percent [34,35]. The corresponding median of the mean diameters were 40 and 39 nm, respectively, and did not differ significantly. For zero, two and four minutes of sonication, the total number of detected aggregates too low (17, 905 and 1220, respectively), to reliably evaluate the median of the mean diameter for these sonication times could not be evaluated reliably. The graphs of Figure 2 indicate that sonication does change the NM studied as the number of smaller particles increases with sonication time, however this article does not consider in any detail the changes introduced by sonication.

To examine the intrinsic properties of silica NM, samples were diluted in double distilled water allowing high adsorption of the fraction of nano-sized particles to the grid surface. For silica NM dispersed in water, fifteen to thirty percent of the grid surface was covered by the silica NM, the particles were homogenously distributed over the grid surface and were well separated with only occasional overlap (Figure 1). The contrast of amorphous silica is caused by thickness contrast and it appears that the clusters of silica are fairly flat.

### Recording, storage and analysis of micrographs

Because of their relatively low molecular mass and amorphous structure, the contrast between silica NM and the background tends to be relatively low when using conventional BF-TEM. The before mentioned combination of a Tecnai G^2^ Spirit TEM (FEI, Eindhoven, The Netherlands) operating at 120 kV equipped with a Biotwin lens configuration and a 4*4 k Eagle CCD camera (FEI, Eindhoven, The Netherlands) allowed however recording images of silica NM in BF mode with a contrast suitable for semi-automatic particle detection and analysis (Figure 1). A complete traceability of information was obtained when storing the micrographs in the dedicated iTEM database.

In the micrographs of the examined NM, aggregates and agglomerates could be detected semi-automatically based on their electron density and analyzed quantitatively. Under the applied imaging conditions the useful range where the particle size can be measured with a precision of 95% [36] contained 95% to 98% of the detected particles. Two to five percent of the detected particles were larger than the upper boundary of the useful range. Hardly any of the detected particles (< 0.1%) were smaller than the lower boundary of the useful range.

Since primary particles in aggregates could not be detected separately, differences in the size of primary particles, as illustrated in Figure 1C and Figure 1D, could not be measured. The raw data resulting from such image analyses consist of 2D matrices containing up to multiple thousands of rows (one for each detected particle) by twenty-four columns (particle identification number and twenty-three measured parameters). The description of the twenty-three parameters considered most relevant are presented in Additional file 1.

### Characterization of silica NM based on quantitative measures

No significant micrograph and no sample effects (P < 0.05) were observed in a non-parametric one-way ANOVA and pairwise comparison with Dunn’s Method (data not shown). In Table 1, Table 2, Table 3 and Table 4 the number of observations (n), the average value (Mean), the standard deviation (SD) and the standard error of mean (SEM) are presented in addition to the largest observation (Max) and the smallest (Min). However, because none of these parameters are normally distributed (P < 0.001) non-parametric estimates of these parameters describe the sample better. These include the median and the 25 and 75 percentiles in Table 1, Table 2, Table 3 and Table 4.

**Table 1:** Descriptive statistics of silica NM-200

**Column**	**n**	**Mean**	**SD**	**SEM**	**Max**	**Min**	**Median**	**25%**	**75%**
Area (nm²)	8005	2112	6730	75	174446	17	438	146	1443
Convex area (nm²)	8005	3385	12076	134	328677	18	553	168	2011
Rectangle max (nm²)	8005	5674	20452	228	565985	30	910	273	3323
Rectangle mean (nm²)	8005	5134	18383	205	503770	26	832	250	3026
Rectangle min (nm²)	8005	4434	15721	175	424206	22	727	220	2623
ECD (nm)	8005	35,7	37,5	0,4	471,2	4,7	23,6	13,6	42,8
Feret max (nm)	8005	56,2	66,4	0,7	883,1	6,0	34,5	18,8	66,0
Feret mean (nm)	8005	47,2	54,9	0,6	717,9	5,1	29,0	15,9	55,4
Feret min (nm)	8005	35,6	41,1	0,4	524,7	3,9	21,9	12,2	42,1
Radius of inner circle (nm)	8005	9,00	5,96	0,06	89,42	1,49	7,47	5,08	11,66
Central distance max (nm)	8005	30,1	36,3	0,4	518,6	2,7	18,2	9,7	35,3
Central distance mean (nm)	8005	18,6	20,4	0,2	266,3	2,1	12,0	6,7	22,0
Central distance min (nm)	8005	6,13	6,55	0,07	120,10	0,07	4,39	2,57	7,36
Diameter max (nm)	8005	56,1	66,4	0,7	883,0	5,6	34,4	18,7	65,8
Diameter mean (nm)	8005	50,0	59,1	0,6	775,1	5,0	30,6	16,6	59,0
Diameter min (nm)	8005	36,8	42,9	0,4	541,0	3,7	22,4	12,3	43,4
Convex perimeter (nm)	8005	153	182	2	2392	14	93	50	181
Perimeter (nm)	8005	254	520	5	12079	15	101	52	235
Aspect ratio	8005	1,556	0,349	0,003	3,607	1,040	1,480	1,298	1,733
Convexity	8005	0,789	0,123	0,001	1,000	0,362	0,803	0,699	0,895
Elongation	8005	1,722	0,508	0,005	5,055	1,000	1,603	1,351	1,968
Shape factor	8005	0,512	0,249	0,002	1,007	0,010	0,516	0,303	0,728
Sphericity	8005	0,414	0,196	0,002	0,989	0,039	0,389	0,258	0,548

**Table 2:** Descriptive statistics of silica NM-201

**Column**	**n**	**Mean**	**SD**	**SEM**	**Max**	**Min**	**Median**	**25%**	**75%**
Area (nm²)	2573	3896	13175	259	420592	35	1021	342	2908
Convex Area (nm²)	2573	6158	21840	430	609588	36	1270	377	4089
Rectangle Max (nm²)	2573	10152	34949	689	898373	52	2039	599	6772
Rectangle Mean (nm²)	2573	9255	32209	634	862557	51	1857	550	6152
Rectangle Min (nm²)	2573	8116	28913	570	826460	46	1638	486	5327
ECD (nm)	2573	50,0	49,5	0,9	731,7	6,7	36,0	20,8	60,8
Feret Max (nm)	2573	77,0	86,0	1,0	1150,0	7,0	51,0	27,0	93,0
Feret Mean (nm)	2573	65,0	71,0	1,0	938,0	7,0	43,0	23,0	79,0
Feret Min (nm)	2573	49,0	55,0	1,0	740,0	4,0	33,0	18,0	59,0
New Radius of Inner Circle (nm)	2573	12,20	7,50	0,10	151,60	2,0	11,0	7,40	15,20
Central Distance Max (nm)	2573	41,7	47,5	0,9	641,9	3,5	26,9	14,2	49,9
Central Distance Mean (nm)	2573	25,8	26,4	0,5	371,8	2,9	18,1	10,3	31,2
Central Distance Min (nm)	2573	8,38	8,90	0,17	200,52	0,03	6,39	3,72	9,98
Diameter Max (nm)	2573	77,0	86,0	1,0	1150,0	7,0	50,0	27,0	93,0
Diameter Mean (nm)	2573	69,0	76,0	1,0	985,0	7,0	45,0	24,0	83,0
Diameter Min (nm)	2573	51,0	57,0	1,0	748,0	4,0	34,0	18,0	61,0
Convex Perimeter (nm)	2573	214	238	4	3139	20	141	75	259
Perimeter (nm)	2573	360	708	13	13479	21	155	76	347
Aspect Ratio	2573	1,529	0,317	0,006	3,388	1,023	1,461	1,296	1,714
Convexity	2573	0,799	0,122	0,002	0,993	0,338	0,812	0,713	0,907
Elongation	2573	1,683	0,457	0,009	4,343	1,000	1,590	1,342	1,924
Shape Factor	2573	0,518	0,259	0,005	1,004	0,013	0,523	0,298	0,747
Sphericity	2573	0,424	0,193	0,003	0,983	0,053	0,395	0,270	0,555

**Table 3:** Descriptive statistics of silica NM-202

**Column**	**n**	**Mean**	**SD**	**SEM**	**Max**	**Min**	**Median**	**25%**	**75%**
Area (nm²)	4248	4039	9319	142	177792	35	1127	422	3335
Convex Area (nm²)	4248	7375	20734	318	445959	37	1536	531	5086
Rectangle Max (nm²)	4248	12683	36710	563	817213	58	2549	874	8562
Rectangle Mean (nm²)	4248	11409	32793	503	737974	53	2305	798	7764
Rectangle Min (nm²)	4248	9785	28110	431	671026	46	2014	697	6673
ECD (nm)	4248	53,2	48,0	0,7	475,7	6,7	37,8	23,1	65,1
Feret Max (nm)	4248	90,0	96,0	1,0	1006,0	7,0	58,0	33,0	107,0
Feret Mean (nm)	4248	74,0	78,0	1,0	865,0	7,0	48,0	28,0	88,0
Feret Min (nm)	4248	55,1	56,7	0,8	675,9	4,1	37,2	21,5	65,8
New Radius of Inner Circle (nm)	4248	11,24	5,97	0,09	51,74	2,09	9,87	6,87	14,05
Central Distance Max (nm)	4248	48,4	52,9	0,8	590,2	3,7	31,0	17,5	57,6
Central Distance Mean (nm)	4248	28,3	27,5	0,4	289,7	3,1	19,3	11,7	34,2
Central Distance Min (nm)	4248	6,94	6,64	0,10	84,15	0,03	5,23	2,86	8,59
Diameter Max (nm)	4248	89,0	96,0	1,0	1006,0	7,0	58,0	33,0	107,0
Diameter Mean (nm)	4248	79,0	85,0	1,0	914,0	7,0	51,0	29,0	94,0
Diameter Min (nm)	4248	57,3	59,8	0,9	714,8	4,2	38,5	22,1	68,3
Convex Perimeter (nm)	4248	245	259	3	2849	21	158	91	291
Perimeter (nm)	4248	468	865	13	17955	21	197	99	453
Aspect Ratio	4248	1,596	0,367	0,005	3,811	1,032	1,518	1,327	1,793
Convexity	4248	0,726	0,128	0,001	0,991	0,302	0,730	0,635	0,823
Elongation	4248	1,805	0,548	0,008	5,474	1,000	1,679	1,403	2,081
Shape Factor	4248	0,386	0,231	0,003	0,966	0,006	0,354	0,192	0,557
Sphericity	4248	0,383	0,193	0,002	0,988	0,033	0,355	0,231	0,508

**Table 4:** Descriptive statistics of silica NM-203

**Column**	**n**	**Mean**	**SD**	**SEM**	**Max**	**Min**	**Median**	**25%**	**75%**
Area (nm²)	4889	3426	8413	120	161619	35	928	362	2740
Convex Area (nm²)	4889	6467	19253	275	454517	37	1314	450	4243
Rectangle Max (nm²)	4889	11063	33198	474	741224	56	2180	740	7163
Rectangle Mean (nm²)	4889	9987	29909	427	692283	53	2001	673	6474
Rectangle Min (nm²)	4889	8598	25731	368	611812	42	1734	597	5586
ECD (nm)	4889	48,5	44,7	0,6	453,6	6,7	34,3	21,4	59,0
Feret Max (nm)	4889	83,0	90,0	1,0	986,0	7,0	53,0	31,0	98,0
Feret Mean (nm)	4889	69,0	74,0	1,0	838,0	7,0	45,0	26,0	81,0
Feret Min (nm)	4889	51,0	54,4	0,7	641,1	4,9	33,5	19,7	60,2
New Radius of Inner Circle (nm)	4889	10,03	5,26	0,07	48,75	1,49	9,27	6,28	12,26
Central Distance Max (nm)	4889	44,7	49,5	0,7	531,1	3,6	28,3	16,4	52,8
Central Distance Mean (nm)	4889	26,1	26,1	0,3	277,2	3,0	17,6	10,9	31,2
Central Distance Min (nm)	4889	6,10	5,96	0,08	76,76	0,02	4,48	2,48	7,60
Diameter Max (nm)	4889	83,0	90,0	1,0	986,0	7,0	53,0	31,0	98,0
Diameter Mean (nm)	4889	73,0	80,0	1,0	880,0	7,0	47,0	27,0	87,0
Diameter Min (nm)	4889	53,3	57,3	0,8	678,8	4,0	34,9	20,3	62,7
Convex Perimeter (nm)	4889	226	245	3	2818	21	147	84	266
Perimeter (nm)	4889	439	839	12	18139	21	182	91	411
Aspect Ratio	4889	1,599	0,357	0,005	3,565	1,039	1,533	1,328	1,794
Convexity	4889	0,717	0,135	0,001	1,000	0,331	0,722	0,622	0,822
Elongation	4889	1,810	0,536	0,007	5,008	1,000	1,700	1,408	2,095
Shape Factor	4889	0,384	0,234	0,003	0,991	0,006	0,354	0,190	0,554
Sphericity	4889	0,379	0,190	0,002	0,981	0,039	0,346	0,228	0,504

PCA of the dataset comprising the twenty-three parameters obtained by quantitative TEM analysis allowed classifying these parameters in three uncorrelated principle components (PC) explaining approximately 93% of the variability in the samples (Additional file 2). Examination of the component pattern profiles of this PCA, given in Additional file 3, for NM-202 shows that PC 1 basically consists of direct size measures and 2D size measurements. The direct size measures include the feret max, feret mean, feret min, central distance max, central distance mean, diameter max, diameter mean and diameter min, the 2D size measurements include area, convex area, rectangle max, rectangle mean, rectangle min, ECD, convex perimeter and perimeter. PC 2 is importantly determined by the aspect ratio, the elongation and the sphericity, which reflect the shape of the particles. PC 3 is mostly determined by the convexity and shape factor, parameters reflecting the surface topology of the particles.

One representative parameter was selected from each of the classifications based on PCA to describe and compare the examined silica NM. The mean diameter was chosen as a size measure, the sphericity was chosen as a shape measure and the shape factor was chosen as a measure for surface topology.

**Figure 3:**
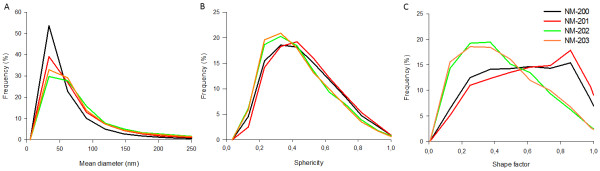
**Number-based distributions of the mean diameter (A), sphericity (B) and shape factor (C) of agglomerates and aggregates of SAS NM.** The frequency of the agglomerates and aggregates of SAS NM: NM-200 (Black), NM-201 (Red), NM-202 (Green) and NM-203 (Orange) are represented as a function of mean diameter, sphericity and shape factor.

Based on the number-based distributions of the mean diameter (Figure 3A) and on the comparison of the medians of the mean diameters (Table 5) of the aggregates and agglomerates, the precipitated NM-200 and NM-201 cannot unambiguously be distinguished from the pyrogenic NM-202 and NM-203. Although the number-based size distribution of NM-200 is different to the curves of NM-202 and NM-203, and its median of the mean diameter is significantly different from that of the pyrogenic NM-202 and NM-203, the number-based size distribution of NM-201 is comparable to the curves of NM-202 and NM-203, and its median of the mean diameter is not significantly different from that of the pyrogenic NM-202 and NM-203.

**Table 5:** Comparison of the characteristics of agglomerates and aggregates of SAS NM

	**Mean diameter (nm)^x^**	**Sphericity ^x^**	**Shape factor ^x^**	**% < 100 nm ^x,y^**
NM-200	31 ± 3 ^a^	0,39 ± 0,01 ^a^	0,51 ± 0,02 ^a^	94 ± 1 ^a^
NM-201	43 ± 4 ^a,b^	0,4 ± 0,01 ^a^	0,56 ± 0,05 ^a^	91 ± 2 ^a,b^
NM-202	53 ± 9 ^b^	0,36 ± 0,01 ^b^	0,35 ± 0,01 ^b^	87 ± 2 ^b^
NM-203	48 ± 4 ^b^	0,35 ± 0,02 ^b^	0,35 ± 0,02 ^b^	88 ± 2 ^b^

^x^ Mean values of medians **±** SD are represented for 3 independent analyses.

^y^ The percentage of particles with a minimal feret diameter smaller than 100 nm is represented.

^a, b^ Different letters indicate significantly different mean values by Kruskal-Wallis One Way Analysis of Variance on Ranks (P < 0,05).

Figure 3B and Figure 3C show that the number-based sphericity and shape factor distributions of the precipitated NM-200 and NM-201 are very similar, as are the corresponding distributions of the pyrogenic NM-202 and NM-203. However, the curves of the precipitated and pyrogenic NM tend to diverge. Table 5 confirms that the median sphericities and shape factors of the pyrogenic and precipitated NM are significantly different, whereas within the precipitated and pyrogenic NM no significant differences were found.

## Discussion

Because of its high resolution, electron microscopy is considered a key method for NM characterization [3,14,38]. The presented methodology complements the visualization and the qualitative description of NM based on representative micrographs. Aggregates and agglomerates of SAS are characterized quantitatively based on threshold based, semi-automatic analysis of BF TEM micrographs.

To characterize a NM, and for *in vivo* and *in vitro* toxicological testing, sonication is recommended as a standard preparatory step to disperse large aggregates and agglomerates [39]. In a pilot experiment, the sonication energy required to prepare a SAS NM sample in its most disperse state was determined as suggested by Powers *et al.*[40] and the conditions for the attachment of particles to the EM-grid were optimized. In our sample preparation, a sonication energy of approximately 2500 MJ/m^3^ was applied.

The general guidelines for image acquisition and analysis proposed by Pyrz and Buttrey [31] were adapted to the analysis of SAS NM. TEM imaging conditions were chosen such that a compromise was reached that combined a sufficient number of particles per image with a resolution providing an acceptable number of pixels per particle, while the useful range contained the large majority of the particles.

The preprocessing of images remains limited - only N x N averaging was essential - and is appropriate for all examined SAS. This avoids loss of information and artifacts associated with significant processing, introducing errors into the analysis [31].

Automation allows measuring multiple and arithmetically complex parameters, described in Additional file 1, simultaneously on high numbers of detected particles. This reduces operator-induced bias and assures a statistically relevant number of measurements avoiding the tedious repetitive task of manual measurement.

Since this method contains no steps that are specific for a certain material, it can readily be adapted to characterize aggregates and agglomerates of a variety of NM, provided that they can be coated quantitatively to the EM-grid and distinguished from the background. For most metal oxides and for metallic NM, the latter poses no problem.

Access to multiple parameters allows selecting the optimal parameter in function of a specific material or purpose as exemplified hereafter. The mean diameter, and feret mean [41,42] are the result of multiple diameters measured under different angles. Therefore, using those means provides a more precise estimate of the size of particles with complex surface topology, like SAS, than using simple parameters, such as feret min, feret max, diameter min and diameter max. The measurement of the equivalent circle diameter (ECD), calculated from the projected surface area, assumes a spheroidal particle morphology like most separation and light scattering based techniques. Hence, comparison of results obtained by techniques such as disc centrifugation and dynamic light scattering and ECD measurements fit each other. To define a material as a NM, the percentage of aggregates smaller than 100 nm can be calculated from the number-based distribution of feret min, an estimate for minimal size in one dimension. In the examined sonicated SAS, these percentages were much higher than 50% (Table 5), defining them as NM according to [2]. Since not the aggregate size, but rather the size of the primary particles has to be smaller than 100 nm, the actual percentage can be assumed much higher. The standard deviation of the percentage of NM smaller than 100 nm ranges from one to 2% and suggests that this method can also be useful in specific cases where, warranted by concerns for environment, health, safety or competitiveness, the number size distribution of 50% may be replaced by a threshold between 1 and 50% [2]. Size measures like the aggregate projected area (area) and the aggregated maximum projected length (feret max) are suitable to assess fractal like NM comprising precipitated and pyrogenic silica NM [19,43]. Combined with the size and overlap coefficient of primary particles, the fractal dimensions can be inferred from these specific aggregate size measures according to [44]. These fractal dimensions are used to explain different phenomena in physics, chemistry, biology and medicine [11]. Van Doren *et al.*[45] investigated the 3D structure of the aggregates of NM-200 and NM-203 by electron tomographic reconstructions. They concluded that the aggregates appear fairly flat, even though structures of primary subunits remain extended in the z-direction, suggesting a limited flexibility of the aggregates. The electron tomographic reconstructions of NM-200 and NM-203 [45] suggest a preferential orientation of the aggregates and agglomerates due to the rolling of aggregates, until a stable position is reached, with a maximal number of contact points [44]. This causes anisotropic effects in the analysis of projected images of particles deposited on a carrier. Such effects are unavoidable in conventional TEM and contribute to larger projected areas and maximum projected lengths. Additionally, in fractal analyses, the number of primary particles and the fractal dimensions may be slightly overestimated [44]. Working in cryo-EM conditions [46], where aggregates are considered to be suspended in vitreous ice, could avoid preferential orientation. This technique requires however, a too high technicity and cost to be practical.

PCA demonstrated that the measured twenty-three parameters could be subdivided objectively into three orthogonal classes representing size, shape and surface topology. Barrett *et al.*[28] proposed the surface texture as a fourth parameter for NM characterization. According to [47], it can be estimated from the fractal dimensions of the particles.

The characterization of a NM by at least one parameter of each of the three classes based on PCA is in line with the guidelines in [3,14,38] that parameters of these classes are essential for the characterization and identification of a NM, *e.g.* in the context of the risk assessment of the application of NM in the food and feed chain. The findings of [16] corroborate this, showing that the size, physical form and morphology parameters determine the access of NM to human cells and cell organelles. In this context, the properties of individual particles measured in two dimensions can be more meaningful, the more because in agreement with [28] subpopulations that cannot be distinguished based on one parameter, can be distinguished based on combinations of parameters for size, shape and surface.

Differences in the production processes of SAS can result in differences in polydispersity, sphericity and shape factor, as illustrated for pyrogenic and precipitated silica NM. Boldrige [19] proposed that for pyrogenic silica the temperature variations occurring near the flame on a microscopic scale result in a greater variability in primary particle size as opposed to precipitated silica where the primary particle size is more homogeneous.

The proposed methodology is developed by studying SAS NM dispersed in water in their most disperse form. It is however generic enough to characterize SAS NM in other media as well, provided that a representative and uniform distribution of the NM on the EM grid can be obtained and that the particles can be distinguished from the background based on their grey values. An adapted sample preparation could be required to obtain this.

For example, SAS in food can be separated from the bulk material by flow field flow fractionation or by extraction procedures [48]. Airborne particles can be sampled and deposited on a grid with a nanoparticle aerosol filter sampler [49]. The effects of salt solutions and proteins on NM aggregation/agglomeration, occurring in *in vivo* and *in vitro* testing [50,51] are also accessible with the described methodology.

Furthermore, the method was successfully applied for the characterization of colloidal silver NM [52] and for the characterization of zinc oxide NM [53], SAS and titanium dioxide NM using the generic NANOGENOTOX dispersion protocol [54], developed for preparation of general batch dispersions for *in vivo* and *in vitro* toxicity testing.

## Conclusion

A quantitative method to assess the characteristics of agglomerated and aggregated NM is presented. BF-TEM combined with systematic random imaging and semi-automatic image analysis allows obtaining an accurate and representative quantification of multiple and arithmetically complex parameters. Access to these parameters allows selecting the optimal parameter in function of a specific material and application. The possibilities of this methodology are explored using precipitated and pyrogenic silica NM as model systems. From number-based size distributions, the percentage of silica aggregates smaller than 100 nm can be quantified. By PCA, the measured twenty-three parameters can be subdivided into three orthogonal classes representing size, shape and surface topology of the NM. Based on this classification, SAS NM could be differentiated according to their production process.

## Competing interests

The authors declare that they have no competing interests.

## Authors’ contributions

PT and JM contributed equally to this manuscript. PT produced most of the data and the illustrations. JM developed the basic concept and the took care of the redaction of the manuscript. ED and EV assisted in the redaction and sample preparation and MF assisted in the adaptation of software and image conversions. YS assisted in the statistical analyses. All authors have read and approved the final manuscript.

## Supplementary Material

Additional file 1 **Table representing the twenty three quantitative parameters and their description as described in the iTEM software.** (PDF 16 kb) Click here for file

Additional file 2 **Table representing the proportion of the eigenvalues of the correlation matrix in each principle component (PC).** (PDF 16 kb) Click here for file

Additional file 3 **Representative component pattern profile of a quantitative TEM analysis of NM-202 categorized into three principle components.** (TIFF 241 kb) Click here for file
