# Supplementary material for: Quantitative characterization of agglomerates and aggregates of pyrogenic and precipitated amorphous silica nanomaterials by transmission electron microscopy
Source: J Nanobiotechnology. 2012 Jun 18;10:24. doi: 10.1186/1477-3155-10-24 (PMC3462150; doi:10.1186/1477-3155-10-24)
Supplement: Additional file 1 — Table representing the twenty three quantitative parameters and their description as described in the iTEM software. (PDF 16 kb) [file 1477-3155-10-24-S1.pdf]

| Measured parameter[a] | Description |
|---|---|
| Area[b,c] (nm²) | Projection area |
| Convex area[b] (nm²) | The area of the convex hull (envelope) bounding the measured object. |
| Rectangle max (nm²) | The area of the biggest rectangle whose sides consist of tangents to the measured object borders. |
| Rectangle mean (nm²) | The area of the mean rectangle whose sides consist of tangents to the measured object borders. |
| Rectangle min[d] (nm²) | The area of the smallest rectangle whose sides consist of tangents to the measured object borders. |
| ECD[e] (nm) | The equivalence refers to the area of the measured object. The ECD is the diameter of a circle that has an area equal to the area of the measured object. |
| Feret max[c] (nm) | The maximum distance of parallel tangents at opposing measured object borders. |
| Feret mean[f] (nm) | The mean distance of parallel tangents at opposing measured object borders. |
| Feret min[c] (nm) | The minimum distance of parallel tangents at opposing measured object borders. |
| Radius of inner circle (nm) | Radius of the maximal circle inside the measured object. |
| Central distance max (nm) | The maximum distance between the center and the border of a measured object. |
| Central distance mean (nm) | The mean distance between the center and the border of a measured object. |
| Central distance min (nm) | The minimum distance between the center and the border of a measured object. |
| Diameter max (nm) | The maximum diameter of a measured object (for angles in the range 0° through 179° with step width 1°). |
| Diameter mean (nm) | The mean diameter of a measured object (for angles in the range 0° through 179° with step width 1°). |
| Diameter min (nm) | The minimum diameter of a measured object (for angles in the range 0° through 179° with step width 1°). |
| Convex perimeter[b] (nm) | The length of the perimeter of the convex hull (envelope) bounding the particle. |
| Perimeter[b] (nm) | The sum of the pixel distances along the closed boundary. |
| Aspect Ratio [g] | The maximum ratio of width and height of a bounding rectangle for the measured object. |
| Convexity [h] | The fraction of the measured object's area and the area of its convex hull. |
| Elongation | The elongation of the measured object can be considered as lack of roundness. It results from the sphericity. |
| Shape factor [i] | The shape factor provides information about the "roundness" of the measured object. For a spherical measured object the shape factor is 1; for all other measured objects it is smaller than 1. |
| Sphericity | Describes the sphericity or 'roundness' of the measured object by using central moments. |

**a These parameters are used in the iTEM software and are described in the iTEM help files**
**b As described in ISO 9276-6:2008**
**c As described in ISO 13322-1:2004**
**d Feret box area[b]**
**e Area equivalent diameter[c]**
**f Angle-average feret diameter**
**g Shape factor[b,c]**
**h Solidity[b]**
**i Form Factor[b]**
