# Supplementary material for: Quantitative characterization of agglomerates and aggregates of pyrogenic and precipitated amorphous silica nanomaterials by transmission electron microscopy
Source: J Nanobiotechnology. 2012 Jun 18;10:24. doi: 10.1186/1477-3155-10-24 (PMC3462150; doi:10.1186/1477-3155-10-24)
Supplement: Additional file 2 — Table representing the proportion of the eigenvalues of the correlation matrix in each principle component (PC). (PDF 16 kb) [file 1477-3155-10-24-S2.pdf]

Product	PC1	PC2	PC3	Cumulative
**NM-200**	73,4 ± 0,7 %	13,5 ± 0,1 %	6,6 ± 0,5 %	93,6 ± 0,3 %
**NM-201**	73,7 ± 0,4 %	13,7 ± 0,1 %	6,8 ± 0,1 %	94,2 ± 0,5 %
**NM-202**	73,5 ± 0,3 %	12,9 ± 0,2 %	6,5 ± 0,2 %	92,9 ± 0,2 %
**NM-203**	74,1 ± 0,4 %	12,9 ± 0,1 %	6,4 ± 0,1 %	93,0 ± 0,3 %
