# Supplementary figures and images for: Quantitative characterization of agglomerates and aggregates of pyrogenic and precipitated amorphous silica nanomaterials by transmission electron microscopy
Source: J Nanobiotechnology. 2012 Jun 18;10:24. doi: 10.1186/1477-3155-10-24 (PMC3462150; doi:10.1186/1477-3155-10-24)

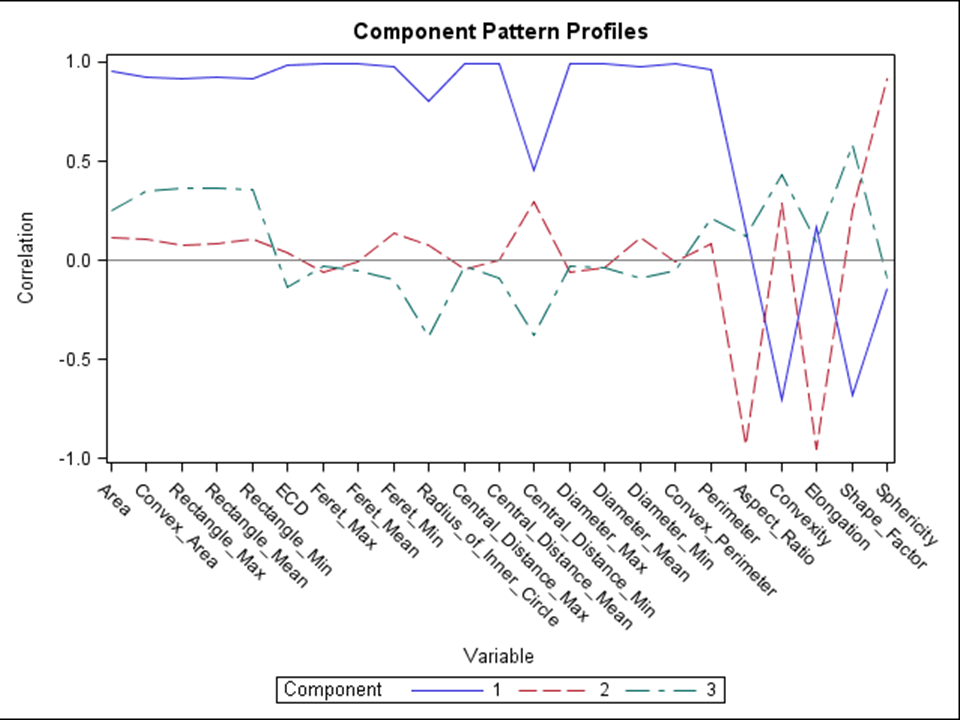

Supplement: Additional file 3 — Representative component pattern profile of a quantitative TEM analysis of NM-202 categorized into three principle components. (TIFF 241 kb) [file 1477-3155-10-24-S3.tiff]
